# Shock-induced nucleation of nanophase Fe-Ni alloy and its implications for interstellar iron reservoirs

**DOI:** 10.3389/fchem.2026.1774797

**Published:** 2026-02-20

**Authors:** Prakash Velampatti Selvaraj, Vijayanand Chandrasekaran

**Affiliations:** 1 Department of Chemistry, School of Advanced Sciences, Vellore Institute of Technology, Vellore, Tamil Nadu, India; 2 Department of Analytics, School of Computer Science and Engineering, Vellore Institute of Technology, Vellore, Tamil Nadu, India

**Keywords:** laboratory astrochemistry, shock-wave, metal vapour chemistry, nanophase Fe-Ni alloy, sample-return missions, cometary dust

## Abstract

Shock waves are ubiquitous in star-forming regions, protoplanetary disks, and cometary environments, yet their role in processing refractory metals remains poorly understood. Here, we show that laboratory shock-tube experiments produce nanophase Fe–Ni alloy from Fe and Ni powders under conditions resembling low-velocity (1–2 km/s) dust-heating shocks in the interstellar medium and cometary comae. The reflected-shock temperature exceeds 6000 K, and pressures reach around 14.5 bar, persisting for about 2–3 ms and completely vapourising the metal powders into an atomic vapour. Subsequent rarefaction drives a catastrophic thermal quench at ∼10^6^ K/s, inducing direct vapour-phase condensation of bcc kamacite (α-Fe‐Ni) without an intervening taenite phase. X-ray diffraction and Rietveld refinement confirm a homogeneous kamacite solid solution, while FESEM reveals octagonal to sub-spherical particles consistent with condensation from transient vapour/melt droplets. HRTEM, SAED, and FFT analyses reveal well-ordered bcc lattices and high densities of dislocations and deformation twins, suggesting rapid quench crystallisation under extreme non-equilibrium conditions. HAADF–STEM and EDS mapping show atomic-scale compositional uniformity, with Fe:Ni ratios closely matching the initial composition. The microstructures, compositions, and sizes of these shock-synthesised nanophase Fe-Ni alloy particles closely resemble nanophase metals observed in GEMS-bearing IDPs and Wild 2 samples, aligning with Ni-enriched metal vapour inferred from Fe I and Ni I detections in cometary comae. Our results demonstrate that transient, low-velocity shocks can produce nanophase Fe–Ni metal with meteoritic and cometary characteristics, establishing a strong mechanistic link between metal vapour chemistry, dust reprocessing, and the formation of nanoscale kamacite in primitive solar system and interstellar materials.

## 1 Introduction

The cosmic inventory of refractory elements, particularly the abundances of iron and nickel, is essential for understanding the thermal and chemical evolution of the Universe (Kimura et al., 2008; Leroux et al., 2000; Nittler and Ciesla, 2016; Reisener and Goldstein, 2003a; Reisener and Goldstein, 2003b). Iron, the most common refractory element, is primarily produced in Type Ia and core-collapse supernovae and is also present in AGB star outflows (Dwek, 2016). However, the physicochemical carriers and their behaviour in the interstellar medium (ISM) and early solar nebulae are poorly understood. Observations with ultraviolet and X-ray telescopes show that most of the iron produced in core-collapse supernovae is in the gas phase within the ISM (Dwek, 2016). Most of the escaped iron is thought to be trapped in interstellar dust, likely as silicates, carbon or composite grains, and metallic phases, and its distribution varies across different astrophysical environments (Dwek, 2016; Kimura et al., 2017).

Observations with ESO’s Very Large Telescope significantly advanced our understanding of metal vapor chemistry on comets at heliocentric distances between 0.68 and 3.25 AU (Guzik and Drahus, 2021; Hutsemékers et al., 2021; Manfroid et al., 2021; Opitom et al., 2021). Neutral iron (Fe I) and nickel (Ni I) emission lines are ubiquitous in 20 solar system comets, yet the surface temperature (150–340 K) is far too low to sublimate refractory minerals (Manfroid et al., 2021). The average Ni I/Fe I ratio is near unity. This higher Ni/Fe ratio suggests sublimation of materials or a sublimation process. Equilibrium thermodynamics and traditional models suggest that these missing vapours could be locked up in refractory mineral phases such as silicates, oxides or sulfides (Manfroid et al., 2021). This challenges the established models, because metallic iron sublimates at around 1200 K, whereas sulfides do so at 600 K. Additionally, the detection of gaseous neutral Ni I in the coma of comet 2I/Borisov (Guzik and Drahus, 2021), together with many-level fluorescence modelling of C/1996 B2 (Hyakutake) (Bromley et al., 2021), suggests volatile, metal-bearing sources, likely short-lived organometallic compounds or metal-bonded polycyclic aromatic hydrocarbons formed via a non-thermal mechanism rather than the sublimation of silicates (Bodewits and Bromley, 2021; Bromley et al., 2021; Manfroid et al., 2021).

To reconcile these observations, cosmochemists rely on the meteoritic record as the empirical basis for understanding the diversity of extraterrestrial materials. Nanophase iron (npFe) and kamacite (α-Fe,Ni) are widespread in primitive chondrites (Kimura et al., 2008; Leroux et al., 2000; Reisener and Goldstein, 2003a; Reisener and Goldstein, 2003b), interplanetary dust particles (IDPs) (Gainsforth et al., 2019; Keller et al., 1979; Zolensky et al., 2006), and lunar soils (Gu et al., 2025; He et al., 2024; Yan et al., 2022; Yin et al., 2025), where they record extreme non-equilibrium processing rather than the coarse equilibrated textures typical of differentiated iron meteorites. Sample-return missions such as Stardust (81P/Wild 2) (Gainsforth et al., 2019; Gu et al., 2025; He et al., 2024; Ishii et al., 1979; Keller et al., 1979; Matsumoto et al., 2024; Shen et al., 2024; Yan et al., 2024; Zolensky et al., 2006), Hayabusa2 (Ryugu) (Kimura et al., 2024; Matsumoto et al., 2024), and Chang’e 5 (Moon) (Gu et al., 2025; He et al., 2024; Shen et al., 2024; Yan et al., 2024; Yin et al., 2025) have significantly advanced the analytical frontier. They have revealed submicrometer Fe-rich particles within lunar impact glasses produced by high-velocity impacts that induce melting, liquid immiscibility, and ultrafast quenching (Gainsforth et al., 2019; Gu et al., 2025; He et al., 2024; Ishii et al., 1979; Keller et al., 1979; Matsumoto et al., 2024; Shen et al., 2024; Yan et al., 2024; Zolensky et al., 2006). Furthermore, these missions demonstrate that shock metamorphism in asteroidal parent bodies can promote recrystallization, lattice distortion, and mosaicism in Fe–Ni alloys—effects that differ from those resulting from simple thermal annealing (Gainsforth et al., 2019; Gu et al., 2025; He et al., 2024; Ishii et al., 1979; Keller et al., 1979; Matsumoto et al., 2024; Shen et al., 2024; Yan et al., 2024; Zolensky et al., 2006).

These nanophase metals are associated with high-energy shock events in the turbulent ISM. Shock processing involves rapid, high-energy events from supernovae, stellar winds, or impacts—capable of briefly (over microseconds to milliseconds) reaching extreme pressures and temperatures. In this extreme environment, refractory materials are vapourised by intense thermal spikes (Jones et al., 1996). As the vapour cools instantaneously, it recondenses into nanophase solids that preserve the imprint of kinetic control. The classical equilibrium condensation models effectively reproduce the mineralogical sequences under slow-cooling nebular conditions. Still, they failed to capture the non-equilibrium behaviour of minerals crystallising under shock-driven processes (Keller and Messenger, 2011).

Laboratory shock-wave experiments are used to address this gap directly, constraining how metal vapour behaves under the relevant conditions. Studies of extraterrestrial samples, especially meteorites, show that shock pressures of about 15–45 GPa induce partial to complete melting and form shock veins in recrystallised metallic phases (Gillet and Goresy, 2013; Xie and Sharp, 2007). However, comparatively low shock-pressure regimes (<10 GPa) have received less attention, particularly regarding vapor-phase processes. Recent analyses of mildly shocked Ryugu asteroid samples show nanophase kamacite without extensive melting of phyllosilicate-rich matrices or dehydration textures (Kimura et al., 2024), underscoring the significance of this comparatively less-explored regime. In many astrophysical environments, particularly within the ISM, low-velocity shock waves are characterised by velocities of the order of 1–2 km/s (Jones et al., 1996; Rudnitskij, 1997). Under such low-velocity conditions, dust grains are subjected to thermal processing rather than destruction or complete evaporation (Biennier et al., 2017). In addition, recent laboratory studies on the rapid formation of interstellar minerals and mineral quantum dots show that intense shock events can melt precursor materials within milliseconds (Roy et al., 2025). These mineral assemblages produced by shocks are particularly significant for interpreting ISM and cometary environments, especially with respect to condensation from the vapour phase.

In this study, we demonstrate that shock-wave-induced nucleation of Fe-Ni alloys occurs from solid-state Fe and Ni powder precursors (<10 μm) under conditions similar to those in turbulent, metal-rich regions of the ISM. The resulting nanophase metals were thoroughly analysed using advanced electron microscopy and spectroscopy techniques. The findings show that nanophase kamacite (α-Fe-Ni) with a composition of Fe∼_0_._90_Ni∼_0_._10_ can nucleate and grow within transient shocks reaching a reflected shock temperature of about 6,000 K and a reflected shock pressure of around 14.5 bar for ∼2 ms. These results substantiate that shock-induced nucleation offers a plausible natural pathway for producing nanophase Fe-Ni metal, which is vital for interpreting *in-situ* observations of metallic nanoparticles in extraterrestrial materials such as lunar regolith and cometary dust, as well as remote sensing detection of gaseous Fe I and Ni I in comets.

## 2 Materials and methods

### 2.1 Materials

Micron-sized, high-purity iron powder (Fe, spherical, average particle size <10 μm, ≥99.9% metals basis; Thermo Scientific) and nickel powder (Ni, average particle size 3–7 μm, 99.9% metals basis; Alfa Aesar) were purchased from certified suppliers and used without further purification. Ultra-high-purity (UHP) argon gas (Ar, 99.999%) and helium gas (He, 99.999%) were supplied by *Rana* Industrial Gases & Products, Chennai. Acetone (analytical grade) was used to clean the apparatus prior to each experiment. Aluminium (Al) diaphragms were custom-fabricated in-house as described in the Methods section.

### 2.2 Methods

#### 2.2.1 Laboratory shock tube setup

**FIGURE 1:**
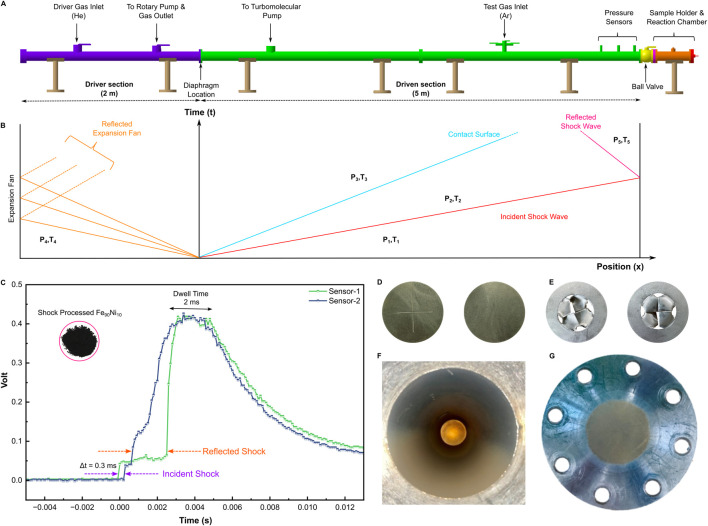
Schematic of the laboratory shock tube and representative pressure trace. **(A)** The shock tube consists of a 2 m high-pressure driver section and a 5.0 m low-pressure driven section separated by an Al diaphragm. **(B)** Typical x-t diagram illustrating shock propagation along the tube. **(C)** Representative oscilloscope traces record the passage of the shock front at two pressure sensors, and the time delay between the upstream and downstream signals is used to determine the shock velocity; Inset in Figure 1C shows the shock-processed sample. **(D,E)** obverse and reverse images of the Al diaphragm before and after rupture. **(F,G)** Photographs showing the shock-processed samples adhering to the reaction chamber and end wall after the experiment.

Shock tubes are widely used in laboratory research to generate controlled shock waves for applications such as combustion studies, chemical kinetics, and aerospace simulations (Bhaskaran and Roth, 2002; Biennier et al., 2017; Chakraborty et al., 2024; Roy et al., 2022a; Roy et al., 2022b). Here, we employ a horizontal 7 m-long stainless-steel tube (Figure 1A), partitioned by an Al diaphragm into a 2 m-high-pressure driver section and a 5 m-low-pressure driven section, with heavy steel end flanges that serve as mechanical dampers. The tube has an internal diameter of roughly 80 mm and an external diameter of about 115 mm. Two piezoelectric pressure transducers (PCB Piezotronics; sensitivity ∼0.5 mV/PSI) were mounted 30 cm apart in the driven section and connected to a digital storage oscilloscope (Tektronix, TDS2014B), and were used to determine the incident shock speed (Supplementary Figure S1).

#### 2.2.2 Sample preparation and loading

Before each experiment, the driven section was evacuated, purged, and then filled with UHP Ar to 0.08 bar. The inner wall of the tube was cleaned with acetone to minimise particulate and organic contamination. Solid precursors, such as Fe and Ni powders, were weighed in the proportions listed in Table 1 and blended to form a homogeneous mixture. Approximately 0.2 g of the mixture was then spread as a thin layer on the sample mount along the inner wall near the closed end of the driven section. After loading the sample, the tube was sealed and backfilled to a base pressure of approximately 10^–2^ mbar, then refilled with UHP Ar to 0.08 bar. A 2.5 mm-thick Al diaphragm (Figure 1D), with precisely machined 0.5 mm grooves separating the driver and driven sections, controlled the shock strength.

**TABLE 1:** Typical experimental shockwave parameters.

Sample No	Experimental condition	Driver gas (He) pressure in bar	Driven gas (Ar) pressure in bar	Velocity (kms^−1^)	*M*	*T(K)*		*P_r_* (bar)
						*T*_*i*_	*T*_*r*_	
1	Fe + Ni (9:1)	44.1	0.08	1.68	5.23	2,813	6,278	14.54

*M*, incident Mach number; *T*_*i*_ and *T*_*r*_, incident and reflected shock temperatures; *P*_*r*_, reflected shock pressure.

To generate the shock, the driver and driven sections were evacuated and flushed with UHP He. The driver section was rapidly pressurised with He at a flow rate (∼1.69 × 10^−3^ m^3^/s) until, at a driver pressure of ∼44 bar, the diaphragm ruptured (Figure 1E), thereby generating an incident shock wave. The rupture allowed the high-pressure He to expand into low-pressure Ar in the driven section, creating a primary shock wave that propagated along the tube and first interacted with the precursor materials. This resulted in an initial shocked state defined by the incident pressure (P_2_) and temperature (T_2_). Upon reaching the end flange, the primary shock wave reflected and propagated back through the already shocked gas. The reflected shock wave re-encountered the sample for a second time, characterised by the reflected pressure (P_5_) and temperature (T_5_). The reflected shock significantly increased the pressure and temperature to levels exceeding those of the primary shock. The key shock-wave parameters, including reflected-shock temperature, pressure, and velocity, were obtained from standard normal-shock relations (Equations 1–4), and the calculated values are summarised in Table 1. The tube remained at a pressure of ∼12 bar for several hours following the shock to allow particle settling. After carefully venting to atmospheric pressure, samples were collected from both the end wall and the inner wall (Figures 1F,G). The overall mass recovery was about 75%, though some loss occurred due to nanoparticle entrainment during depressurization. The resulting shock-processed samples were carefully characterised and analysed using powder X-ray diffraction (pXRD) with Rietveld refinement, advanced electron microscopy, and elemental mapping to assess phase purity, nanoscale particle size, and detailed microstructural features of the Fe–Ni alloy nanoparticles.

The primary shock velocity, $v_{s}$ , was determined experimentally using the time-of-flight method. Piezoelectric pressure transducers located at fixed intervals *L* (300 mm) along the shock tube recorded the shock arrival times (Figures 1B,C). The velocity was calculated as $v_{s}=L/\Delta t$ , where Δt is the transit time between sensors. The incident Mach number, *M*, was subsequently defined relative to the pre-shock sound speed, $c_{1}$ :

$$M=\frac{v_{s}}{c_{1}}=\frac{v_{s}}{\sqrt{\gamma RT_{1}}}$$ (1)

where γ is the adiabatic index, *R* is the specific gas constant, and *T*_*1*_ is the initial temperature of the test gas.

#### 2.2.3 Reflected shock conditions

The thermodynamic state behind the reflected shock wave was calculated by enforcing the boundary condition of zero particle velocity at the shock tube end-wall ( $v_{5}=0$ ). Assuming an ideal gas, the pressure ratio across the reflected shock, $p_{5}/p_{2}$ , is determined by the strength of the incident shock, $p_{2}/p_{1}$ :

$$\frac{p_{5}}{p_{2}}=\frac{\left(3\gamma -1\right)\left(p_{2}/p_{1}\right)-\left(\gamma -1\right)}{\left(\gamma -1\right)\left(p_{2}/p_{1}\right)+\left(\gamma +1\right)}$$ (2)

The total pressure jump relative to the initial state $\left(p_{5}/p_{1}\right)$ can be expressed directly in terms of the incident Mach number, *M*:

$$\frac{p_{5}}{p_{1}}=\left[\frac{2\gamma M^{2}-\left(\gamma -1\right)}{\gamma +1}\right]\left[\frac{\left(3\gamma -1\right)M^{2}-2\left(\gamma -1\right)}{\left(\gamma -1\right)M^{2}+2}\right]$$ (3)

The reflected temperature, $T_{5}$ , was derived from the equation of state using the calculated pressure ratios:

$$\frac{T_{5}}{T_{1}}=\frac{T_{5}}{T_{2}}\times \frac{T_{2}}{T_{1}}=\left(\frac{p_{5}}{p_{2}}\right)\left(\frac{\frac{\gamma +1}{\gamma -1}+\frac{p_{5}}{p_{2}}}{1+\frac{\gamma +1}{\gamma -1}\frac{p_{5}}{p_{2}}}\right)\times \frac{T_{2}}{T_{1}}$$ (4)

P_1_ and T_1_ are the initial pressure and temperature of the driven sections, and they are 0.08 bar and 300 K, respectively.

#### 2.2.4 Powder X-ray diffraction (XRD) and rietveld refinement analyses

The XRD patterns of the recovered samples were recorded on a Bruker D8 Advance diffractometer using Cu Kα radiation (λ = 1.5406 Å). Scans were collected over the range 5°–90° with a step size of 0.02° to obtain high-resolution data for phase and crystallographic analysis. The Rietveld refinement of the diffraction data was performed using the GSAS II software package.

#### 2.2.5 Field emission scanning electron microscopy (FESEM) analyses

The morphology and composition of the shock-treated samples were examined using a Thermo Fisher FEI QUANTA FEG field-emission SEM equipped with an Oxford Instruments X-max silicon drift EDS detector. A small portion of the sample was mounted on conductive, vacuum-compatible carbon tape and sputter-coated with a thin gold (Au) layer to enhance surface conductivity and prevent charging of the magnetic lens prior to imaging. Secondary electron images (SE) were captured in high-vacuum mode at 20 kV with a working distance of 11.5 mm. They were complemented by EDS point analyses and two-dimensional elemental maps to characterise the distributions of Fe and Ni.

#### 2.2.6 Transmission electron microscopy (TEM) analyses

The nanoscale morphology and composition of the Fe-Ni alloys were analysed by TEM using a JEOL JEM-F200 operated at 200 kV, equipped with dual large-area silicon drift detectors (SDDs) to enable highly sensitive EDS analysis. The structural and compositional characterisation employed high-resolution TEM imaging, selected-area electron diffraction (SAED), high-angle annular dark-field scanning TEM (HAADF-STEM), and EDS mapping to resolve the crystal structure, defect features, and nanoscale Fe-Ni distributions.

## 3 Results

### 3.1 Structural characterisation and phase identification

**FIGURE 2:**
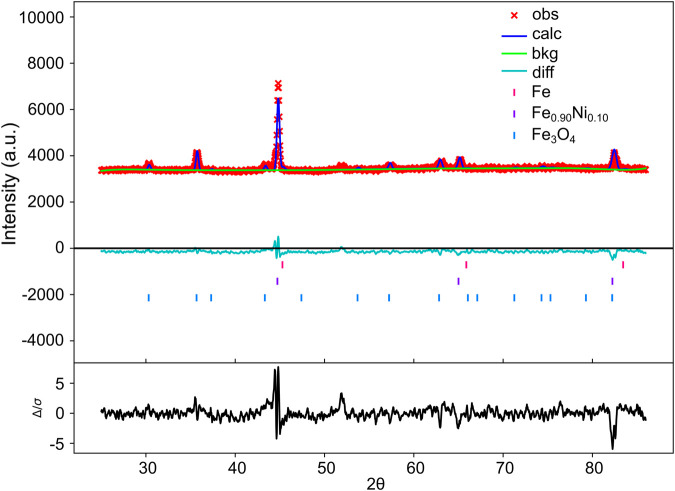
Structural and phase analysis of shock-synthesized kamacite. Rietveld refinement plot of the kamacite sample, showing observed intensities (red cross mark), calculated profile (blue line), and difference curve (cyan line); tick marks denote Bragg peak positions.

The XRD pattern of pure iron prior to shock exposure exhibits a body-centered cubic (bcc) structure under ambient conditions (Supplementary Figure S2). In contrast, the recovered shock-processed Fe-Ni powder shows diffraction peaks corresponding to the (110), (200), and (211) planes, indicative of bcc kamacite (ICDD 00-037-0474) within the Im-3 m space group (No. 229), as illustrated in Figure 2. No indications of phase segregation into Fe-rich or Ni-rich regions were observed, and diffraction peaks associated with austenite/taenite (γ-Fe, Ni, face-centered cubic) or other high-pressure polymorphs were not present. A slight shift in all three planes, especially the (110) peak position (44.86°–44.82°), along with reduced intensity relative to pure iron (Supplementary Figure S2B), suggests lattice strain and substitution effects due to approximately 10% Ni incorporation. Notably, fcc taenite is absent; although equilibrium thermodynamics suggest fcc stabilization in Fe-Ni systems, the rapid cooling rates inherent to shock processing (∼10^6^ K/s) appear to suppress the bcc-to-fcc transition. Secondary peaks correspond to surface magnetite formed through oxidation.

Rietveld refinement was performed using GSAS-II software, confirming the structural integrity of the kamacite phase. The model achieved convergence with high accuracy (Figure 2), exhibiting a weighted R-factor (wR) of 1.696, a goodness of fit (GOF) of 1.00 and χ^2^ of 1.00. These results validate the formation of a homogeneous solid solution with the target stoichiometry.

### 3.2 Field emission scanning electron microscopy (FESEM) morphology and growth dynamics

**FIGURE 3:**
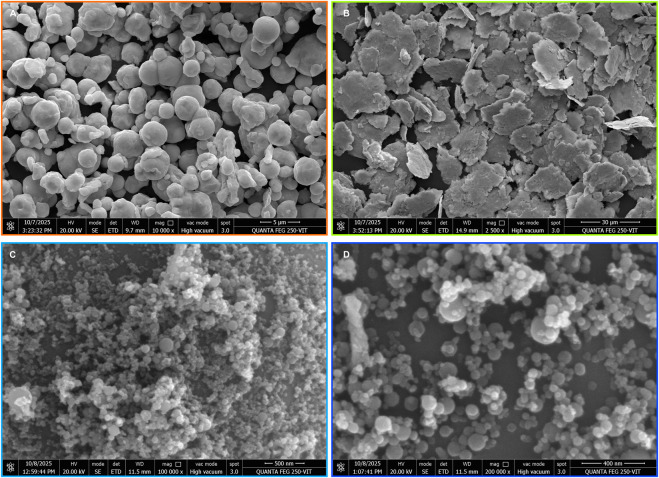
FESEM images of pure Fe, pure Ni, and shock-synthesised kamacite. **(A,B)** Secondary electron micrographs of the pure Fe and Ni precursors. **(C,D)** Secondary electron images of kamacite grains, including aggregates that form loose clusters; the grains exhibit angular (octagonal) to sub-spherical morphologies with well-defined phase boundaries.

Secondary electron (SE) images of the starting materials, such as pure Fe and Ni powders (Figures 3A,B), and the corresponding EDS analyses, are provided in the Supplementary Figures S3,S4. The Fe powders consist of spherical particles, whereas Ni powders appear as flakes with sizes <10 µm. The shock-processed Fe-Ni sample listed in Table 1 was examined using FESEM. SE images of shock-processed (initial 9:1 Fe: Ni ratio) with higher reaction efficiency (Figures 3C,D) mainly consist of kamacite in octagonal to sub-spherical shapes, often loosely clustered—an attribute of condensation from a fluid phase (vapour or melt droplets), where minimisation of surface tension drives the spherical shape prior to solidification.

EDS mapping demonstrates homogeneous Fe and Ni distributions at the submicrometer scale (Figure 5F), with no visible compositional gradients or core-rim structures—unlike kamacite in slowly cooled meteorites. Quantitative point analyses reveal octagonal particles dominated by Fe, with smaller amounts of Ni and trace surface oxygen from minimal post-shock oxidation (S. Figures 5B–D). Particle interiors do not show oxygen enrichment, aligning with the XRD results and suggesting reducing shock conditions that prevented FeO or NiO formation.

### 3.3 High-resolution TEM, STEM-BF, HAADF with EDS mapping

**FIGURE 4:**
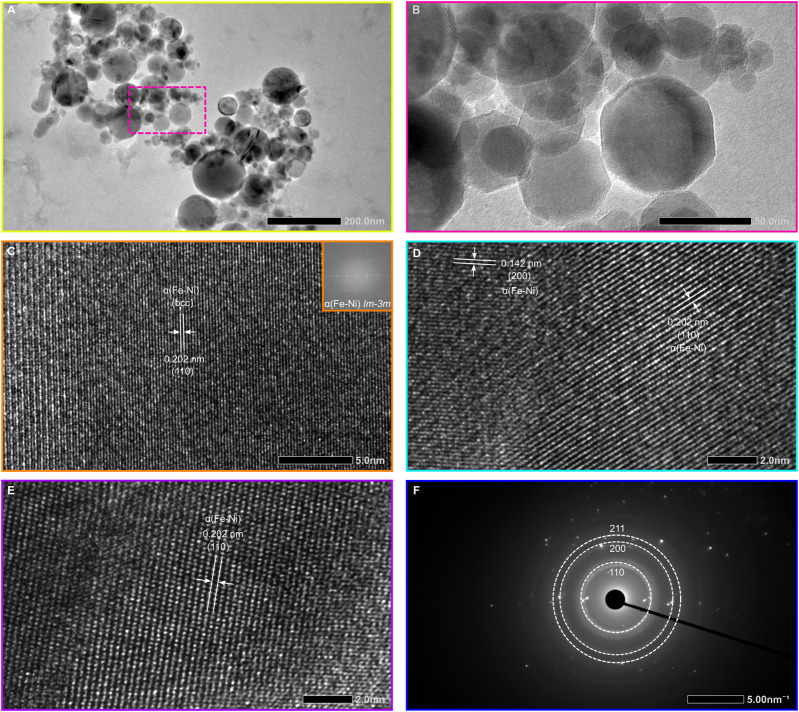
TEM, High-Resolution TEM, SAED pattern of shock-synthesized kamacite. **(A,B)** HRTEM image of post-shock samples showing kamacite with octagonal to sub-spherical morphologies, indicative of condensation from a melt/vapor phase. **(C–E)** High-resolution TEM image illustrating lattice fringes characteristic of the body-centered cubic structure of kamacite (space group Im-3 m). **(F)** Selected-area electron diffraction (SAED) pattern from a single grain, confirming the crystalline structure; prominent reflections are indexed to the (110), (200) and (211) planes.

**FIGURE 5:**
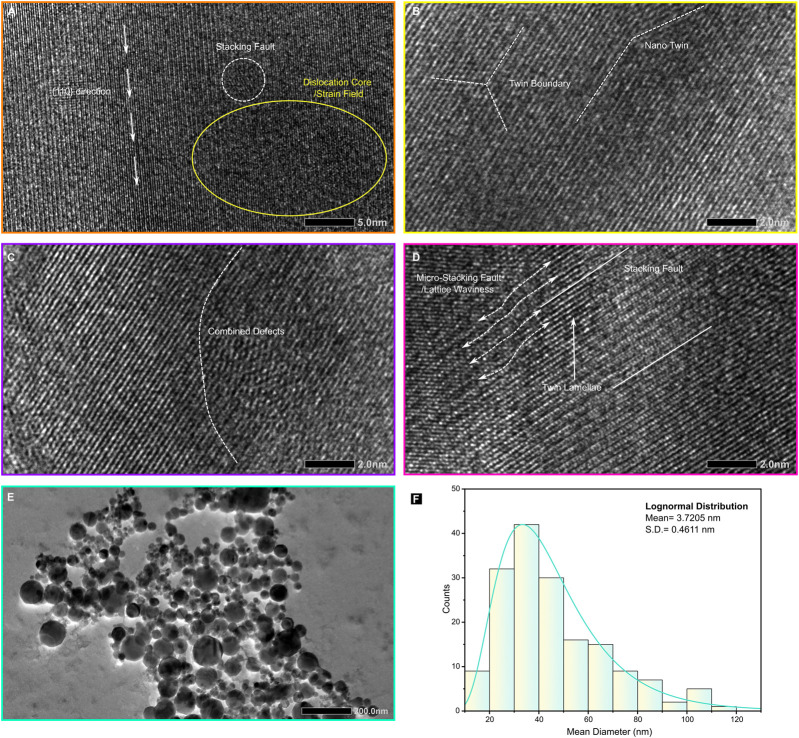
Atomic-scale defects and particle size distribution of shock-synthesized kamacite. **(A)** High-resolution TEM micrograph revealing a dislocation core (yellow circle), identified by the termination of lattice fringes, and stacking faults exhibiting lateral atomic displacement. **(B)** Atomic-resolution image of a twin boundary (white dashed line) demonstrating mirror symmetry between adjacent crystallites. **(C,D)** The coexistence of these defects highlights the interaction between dislocation slip and twinning during shock compression. **(E,F)** Particles selected for analysis and the histogram of kamacite particle size distribution fitted with a log-normal curve; N = 168 particles analyzed.

High-resolution transmission electron microscopy (HRTEM) imaging at 200 kV provides detailed insights into the internal structure of individual Fe–Ni alloy nanoparticles. The images reveal well-defined crystal lattices with interplanar spacings of 2.02 Å and 1.43 Å, corresponding to the {110} and {200} reflections of body-centered cubic (bcc) α-Fe-Ni (kamacite) (Figure 4D). The grains exhibit a high density of structural defects, including dislocations, stacking faults, twin boundaries, and complex defect interactions (Figures 5A–D), with (110) lattice fringes predominantly observed (Figures 4C–E). No indication of magnetite or other Fe–Ni oxide phases was observed, indicating that the nanoparticles largely preserve a metallic state under the shock-synthesis conditions.

Atomic-number-sensitive contrast in HRTEM further reveals nanoscale compositional heterogeneity, with bright regions indicating metallic Ni-rich domains (Figure 4E) embedded within the Fe-rich matrix. Detailed defect analysis reveals that dislocation cores (yellow solid circle) are frequently associated with extensive planar faults and nanotwin lamellae (Figure 5A), suggesting that full dislocation dissociation functions as the primary strain-relief mechanism during rapid quenching. Pronounced lattice distortions (Figure 5D), such as waviness and micro-stacking faults, indicate residual elastic strain and the generation of geometrically necessary dislocations as the crystal lattice contracts under steep thermal gradients.

Nanoscale deformation twins on {110} and {200} planes (Figure 4D), rather than the more commonly observed {112} plane in bulk bcc Fe, indicate that the Fe-Ni alloy underwent intense solid-state deformation under high stress during shock-induced nucleation and cooling. These microstructural features indicate a hardened, defect-rich kamacite formed under extreme non-equilibrium conditions. Fast Fourier Transform (FFT) analysis (inset in Figure 4C) shows that the features closely resemble those of spherical nanophase kamacite inclusions embedded in the matrix of the Martian chassignite NWA 2737 (Vandemoortele et al., 2007). In NWA 2737, the origin of nanophase metallic kamacite has been linked to intense shock metamorphism occurring at very high temperatures and pressures over short time scales, conditions broadly comparable to those produced in the present laboratory shock-wave experiments (Vandemoortele et al., 2007). The SAED pattern of the nanophase Fe-Ni alloys shows concentric diffraction rings consistent with the bcc kamacite (α-(Fe, Ni)) structure (Figure 4F), and these rings are indexed within the Im-3 m space group (No. 229). The measured d-spacing matches exactly the lattice parameters derived from X-ray diffraction, confirming structural consistency across different scales. No extra reflections, diffuse scattering, long-range ordering, or secondary phases were observed.

In bright-field TEM mode (Figure 5E), the Fe–Ni nanoparticles exhibit predominantly octagonal-to-near-spherical morphologies, with diameters ranging from 20 to 200 nm. A log-normal function better describes the Fe-Ni nanoparticle size distribution than a Gaussian, a signature of multiplicative growth in a non-equilibrium regime. This yields a mean diameter of about 33 nm (Figure 5F), consistent with rapid nucleation followed by diffusion-limited coalescence. The dominance of octagonal particles (Figures 4A,B; Supplementary Figure S6) is consistent with rapid condensation of post-shock Fe–Ni vapour, where surface tension and anisotropic surface energies drive facet development before the droplets fully solidify. The observed spread in particle diameters can be explained by spatial variations in the local vapour-phase kamacite density, together with subsequent coarsening through agglomeration and Ostwald ripening under residual thermal gradients.

**FIGURE 6:**
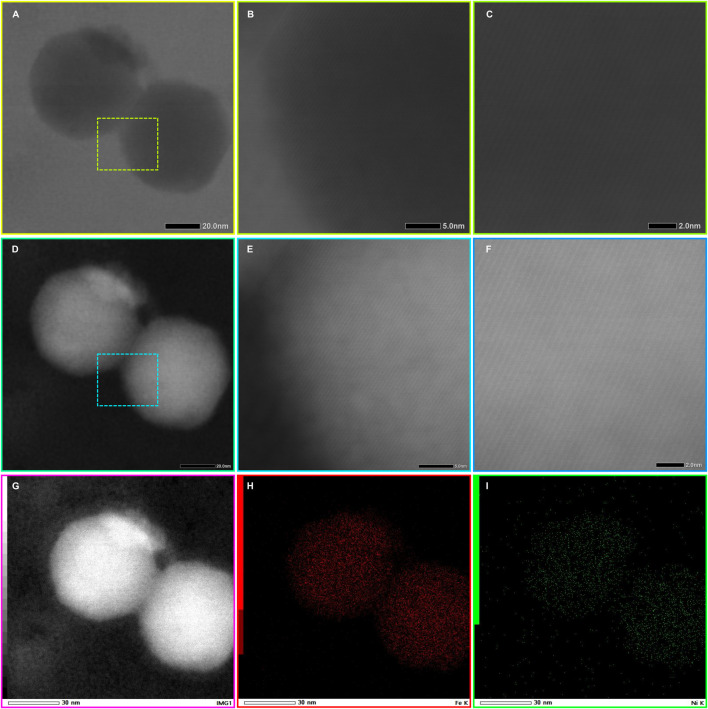
STEM-BF, HAADF imaging, and elemental mapping of nanophase kamacite. **(A–C)** STEM bright-field (BF) images displaying the overall morphology of a representative kamacite grain. **(D–F)** High-angle annular dark-field (HAADF) images emphasising atomic number contrast within the same region and clear lattice fringes along the {110} planes, indicating a homogeneous Fe–Ni distribution at the atomic scale **(G–I)** HAADF image and associated Fe (red) and Ni (green) elemental maps.

**FIGURE 7:**
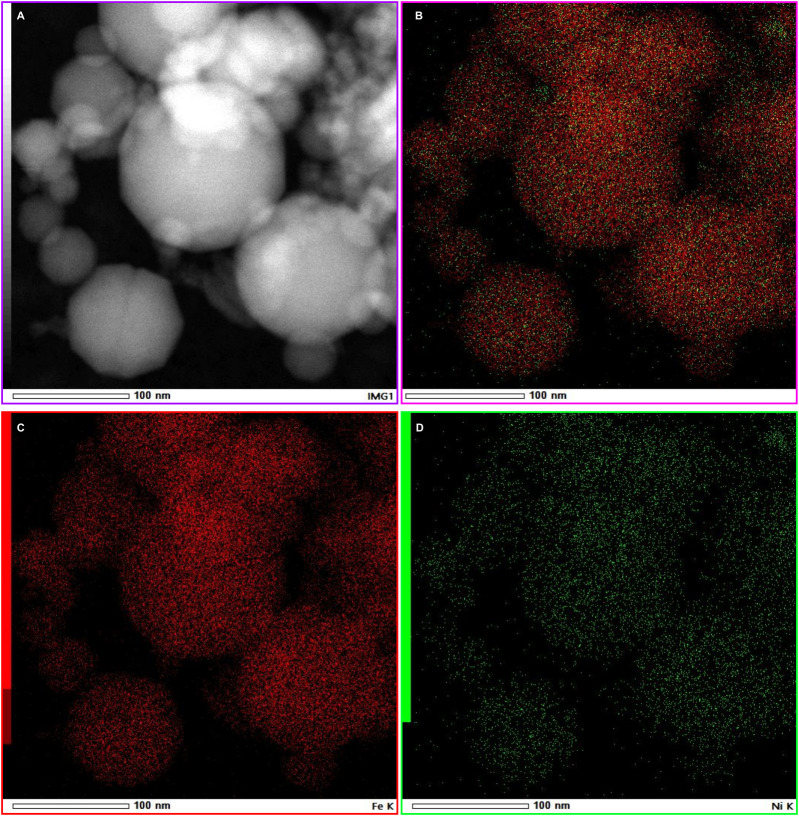
STEM-HAADF imaging and elemental mapping of nanophase kamacite cluster. **(A)** HAADF image with **(B)** showing the combined overlay that reveals Fe/Ni zoning within the kamacite particles. **(C,D)** corresponding Fe (red) and Ni (green) elemental maps.

The bright-field (Figures 6A–C) and high-angle annular dark-field STEM (HAADF-STEM) was used to obtain Z-contrast (atomic-number-sensitive) images of the Fe–Ni nanoparticles (Figures 6D–F). The HAADF images show uniform intensities within individual grains and well-defined lattice fringes along {110} planes (Figures 6E,F), consistent with a chemically homogeneous Fe-Ni solid solution at the atomic scale. Corresponding EDS elemental maps (Figures 6G–I; Figures 7A–D), acquired across multiple grain interiors and boundaries, reveal no detectable Ni enrichment or depletion, indicating that rapid shock quenching suppressed diffusion-controlled partitioning and preserved a uniform composition. Quantitative EDS measurements yield average atomic fractions of Fe = 90.52 ± 0.44% and Ni = 9.48 ± 0.44% (Supplementary Figures S7,S8), values that closely align with the initial composition and are consistent with X-ray diffraction (XRD) results.

## 4 Discussion

Iron-nickel alloys are fundamental building blocks of planetary materials. Yet their formation pathways under the low-pressure, low-temperature conditions typical of the ISM and the outer solar system remain unresolved. Kamacite (α-(Fe, Ni) containing 4–7.5 wt% Ni) is the dominant metallic phase in many extraterrestrial materials. In typical metallurgical processes and during slow cooling in differentiated asteroid cores, the evolution of Fe-Ni alloys is largely governed by the Fe-Ni phase diagram. Sintering mixed Fe and Ni powders at temperatures of about 1,000 °C–1,250 °C promotes diffusion-controlled formation of taenite (γ-Fe, Ni), a nickel-rich fcc phase stabilised at elevated temperatures (Singh et al., 2018). As the alloys cool very slowly, taenite partially transforms into kamacite (α-(Fe, Ni)), a bcc phase, producing the distinctive Widmanstätten pattern in iron meteorites (Yang and Goldstein, 2005). The crystallographic relationship between kamacite and taenite commonly follows Kurdjumov–Sachs or Nishiyama–Wassermann orientation relationships. The associated phase transformation can proceed over millions of years (cooling rates of 1–100 K Myr^-1^), allowing coarse, chemically zoned kamacite-taenite intergrowth to form in octahedrites (Yang and Goldstein, 2005). Recent advances in mapping the Fe-Ni phase diagram through *ab initio* modelling, combined with diamond-anvil cell experiments, provide vital insights into both ambient and planetary core conditions (Sun et al., 2024; Torchio et al., 2020; Wei et al., 2025). The bcc solid solutions dominate at core-like pressures above about 330 GPa and >6000 K, with Ni alloying stabilising the bcc structure and elevating the melting curve (Sun et al., 2024; Torchio et al., 2020; Wei et al., 2025). These high-pressure and high-temperature phase relations help contextualise the prevalence of bcc Fe-Ni observed in rapidly quenched samples formed at far lower pressures.

Compared with slow cooling over millions of years in planetary cores, shock processing imposes extreme kinetic constraints that fundamentally alter the evolution of the Fe-Ni phase. In the present experiment, reflected shocks heat the sample to peak temperatures of >6000 K at reflected pressures of about 14.5 bar for only 2–3 milliseconds. The sample is subsequently cooled at rates of about 10^6^ K/s. Under such nonequilibrium conditions, the thermodynamic path through the Fe-Ni phase diagram is effectively near-vertical, thereby narrowing the time-temperature window in which solid-state diffusion-controlled transformations and exsolution normally occur. This rapid quenching regime favours direct condensation of bcc kamacite nanoparticles from the vapour phase, bypassing the usual fcc (taenite) → bcc (kamacite) transformation sequence and suppressing large-scale chemical zoning. The resulting fine-grained bcc metal microstructures closely resemble those documented in shock-metamorphosed meteoritic metals, where transient high-temperature, high-pressure pulses and rapid cooling also produce nonequilibrium kamacite textures.

The nanophase Fe-Ni alloy produced in these shock experiments shows striking similarities to metal grains found in the most primitive extraterrestrial materials (Vandemoortele et al., 2007). Cometary dust contains abundant amorphous silicates known as GEMS (Glass with Embedded Metal and Sulfides), submicron particles regarded as essential constituents of the outer solar system (Bradley, 1979; Keller and Messenger, 2011). The origin of GEMS remains debated: one view interprets them as chemically homogenised, irradiated interstellar grains later incorporated into the solar system (Bradley, 1979), whereas another favours formation as non-equilibrium condensates in the early solar nebula (Keller and Messenger, 2011). Isotopic studies indicate that only a small percentage (1%–6%) can be confidently identified as surviving presolar circumstellar grains (Keller and Messenger, 2011). In GEMS, nanophase Fe-Ni metals, such as kamacite, are thought to act as nucleation centres around which the amorphous silicate matrix forms the bulk of GEMS (Keller and Messenger, 2011). Rare instances of GEMS with crystalline kamacite cores support this hypothesis, implying that metallic seeds served as substrates for subsequent silicate accumulation. In chondritic porous interplanetary dust particles (IDPs), kamacite inclusions, typically 5–50 nm across, are common, with compositions in the range Fe_0_._93-0_._97_Ni_0_._03-0_._07_ (Keller and Messenger, 2011). The shock-synthesised kamacite reported here has a slightly larger mean size (∼33 nm) and is marginally more Ni-rich, yet remains within the compositional and size range observed for natural kamacite in GEMS-bearing IDPs.

Bradley and co-workers argued that GEMS are ancient, likely presolar materials that largely escaped substantial parent-body thermal metamorphism (Bradley and Dai, 2004). Their embedded metal grains show no evidence of prolonged annealing and instead point to rapid, strongly non-equilibrium formation histories (Bradley, 1979; Ishii et al., 1979). The high dislocation densities, deformation twins, and compositional homogeneity documented in the shock-synthesised kamacite closely parallel the microstructural traits of GEMS-hosted metals, indicating that both may have formed through analogous rapid processes, such as vapour-phase condensation or quench crystallisation from a transient melt.

The Fe-Ni powders used in this study have particle sizes <10 µm and serve as the starting material. This size range was chosen to approximate the dominant fine fraction of lunar-type regolith: particle-size analyses of Chang’E5 soils show that 95% of grains by number fall between 1.40 and 9.35 µm, indicating that micron-scale particles constitute the primary dispersed population on airless planetary surfaces (He et al., 2024; Li et al., 2022). These fine grains, which host nanophase Fe and Fe-Ni-S within impact-melt glass and agglutinates, are the primary carriers of space weathering signatures, including the Moon’s optical darkening and reddening (Gu et al., 2025; Moretti et al., 2005; Pieters and Noble, 2016). From a shock-physics perspective, micron-scale grains are large enough to retain heat and form melt pockets under brief, high-temperature pulses, yet small enough to be heated quasi-uniformly during a single shock event, as demonstrated by *in situ* micrometeoroid-analogue heating experiments on pentlandite that produce Fe-Ni whiskers and S-depleted rims on micron-scale grains (Thompson et al., 2025). In our experiments, the primary and reflected shocks together impose a two-step thermal history that efficiently vapourises and recondenses the material into kamacite nanoparticles. The incident shock first compresses and heats the Ar gas to about 2800 K while accelerating it to a post-shock velocity of ∼1.68 km/s, leaving much of the energy in bulk motion rather than internal heating. When this supersonic flow impinges on the end flange, a reflected shock wave forms and propagates upstream, decelerating the gas and converting kinetic energy into internal energy, thereby raising the temperature to ∼6000 K. Under these extreme conditions, refractory Fe and Ni, with boiling points near 3134 K and 3003 K, respectively, undergo partial melting during the primary shock and then rapid, nonequilibrium evaporation during the reflected shock pulse. The combination of rapid heating and subsequent quenching favours direct vapour-phase condensation of bcc kamacite nanoparticles, bypassing diffusion-controlled transformations and yielding particle sizes, compositions, and a defect-rich microstructure comparable to those inferred for kamacite in GEMS-bearing cometary and interplanetary dust.

NASA’s Stardust mission’s return of samples from comet 81P/Wild 2 provides additional constraints, as they contain nanophase Fe-Ni metal similar to that seen in cometary IDPs, although many grains show modifications associated with hypervelocity capture in silica aerogel (Ishii et al., 1979; Keller et al., 1979; Zolensky et al., 2006). Where minimally altered metal is preserved, compositions cluster around Fe_0_._90_Ni_0_._10_ indistinguishable from our synthetic kamacite and systematically offset from solar Fe_0_._93_Ni_0_._07_, reinforcing the link between experimental products and cometary metals.

The consistent Ni enrichment relative to solar abundances probably reflects vapor-phase fractionation processes. Observations of cometary comae report Ni/Fe ratios near unity, roughly an order of magnitude above solar, indicating that Ni can be strongly overrepresented in the gas phase (Manfroid et al., 2021). Experiments and models of metal vapor condensation under reducing, moderately supersaturated conditions show that Ni nucleates more easily than Fe, offering a natural explanation for the observed enrichment when kamacite condenses from such vapors (Bromley et al., 2021).

The detection of neutral Fe I and Ni I emissions in comets at heliocentric distances of one to three AU, where surface temperatures are only ∼150–340 K, rules out conventional sublimation of refractory minerals (stability >1,200 K) or metallic Fe–Ni (melting ∼1,400–1,500 K) as the dominant source (Guzik and Drahus, 2021; Hutsemékers et al., 2021; Manfroid et al., 2021). Yet production rates of ∼10^22^–10^23^ atoms s^-1^ for Fe and Ni imply substantial metal release into the coma, pointing instead to mechanisms such as non-thermal desorption, chemical erosion, transient grain heating, superheating of submicron particles, impact vaporization reaching ∼10^7^ K, or photodissociation of volatile metal-bearing species such as Fe(CO)_5_ and Ni(CO)_4_ (Guzik and Drahus, 2021; Hutsemékers et al., 2021; Manfroid et al., 2021).

Among these possibilities, the organometallic hypothesis is particularly attractive because Fe(CO)_5_ and Ni(CO)_4_ have sublimation temperatures only slightly above that of CO_2_ (∼150–200 K), consistent with metal release far from the Sun, and Ni(CO)_4_ sublimates roughly ten times faster than Fe(CO)_5_ near 300 K, naturally producing Ni-rich gas (Bodewits and Bromley, 2021; Manfroid et al., 2021). However, metal carbonyls have not yet been directly detected in comets and their formation pathways remain uncertain, motivating an alternative picture in which metal atoms liberated by any of the above mechanisms recondense as nanophase kamacite when local vapor densities and cooling rates are favorable, with kinetic effects during condensation preferentially incorporating Ni.

The radial behaviour of Fe and Ni emissions also constrains the nature of their parent species: a 1/ρ dependence of the brightness profile implies short-lived parents with destruction scales on the order of the nucleus size, and Haser modelling yields parent scales of about 170 km at 1 AU for Ni-bearing species, corresponding to lifetimes of roughly 140–600 s (Guzik and Drahus, 2021). These lifetimes are comparable to the effective interaction time in the shock experiments once differing expansion speeds are taken into account, with shock-processed material expanding at about 1.68 km/s in the laboratory versus around 0.5 km/s in typical cometary comae (Guzik and Drahus, 2021), suggesting that broadly similar kinetic and thermal regimes may operate in both environments.

Astronomical observations of infrared spectra of dust in supernova remnants and at shocked molecular cloud interfaces show contributions from both amorphous silicates and crystalline metal oxides (Rho et al., 2008), indicating ongoing dust reprocessing in these high-temperature regions. If metallic Fe-Ni nanoparticles form there and subsequently oxidise, they could plausibly evolve into the meta-oxide populations responsible for part of the observed infrared emission features (Bouvis et al., 2025). Conversely, in environments that remain sufficiently reducing, some fraction of these metal grains may avoid oxidation, survive as zerovalent Fe-Ni, and later be incorporated into nascent planetary systems as refractory metallic components (Bouvis et al., 2025).

Finally, the characteristic size range of the shock-synthesised nanophase kamacite grains (tens of nanometres) places them in a regime where radiation pressure can strongly influence their dynamics. For such sub-micron particles, the ratio of radiation pressure to stellar gravity can approach or exceed unity, leading to efficient outward acceleration and potential removal from circumstellar regions, preferentially injecting them into the ISM (Burns et al., 1979). This could establish a circulation pattern where metal nanoparticles formed in star-forming regions are expelled into the ISM, mix with gas and dust over gigayear timescales, and ultimately recycled into subsequent generations of protoplanetary disks, where they may reappear as GEMS-like inclusions and related metal phases.

## 5 Conclusions

Our shock-wave synthesis of nanophase Fe-Ni alloy at millisecond timescales, combined with recent astronomical detections of atomic Fe I and Ni I vapors in comets and comprehensive characterization of the synthetic products, establishes a plausible formation pathway for metal nanoparticles in various cosmic environments. The key findings of this study are:

1. Nanophase kamacite with a composition of Fe_∼0.90_Ni_∼0.10_, grain sizes of 20–200 nm (mean 33 nm), and a bcc crystal structure forms within milliseconds under reflected shock pressure of 14.5 bar and temperatures of ∼6000 K, demonstrating that extended thermal processing is not necessary for metal alloy formation. High dislocation densities, deformation twins, compositional homogeneity, and the lack of chemical ordering in the shock-synthesised kamacite suggest it formed under far-from-equilibrium conditions with ultrarapid cooling (∼10^6^ K s^-1^). This is consistent with vapour condensation or quench crystallisation.
2. By closely matching the sizes, compositions, and defect textures of kamacite in GEMS, chondritic-porous IDPs, and minimally altered cometary particles, these experiments provide a solid physical mechanism for producing nanophase Fe–Ni metal in astrophysical shocks. The correspondence between experimental conditions and inferred interaction timescales in cometary comae suggests that similar transient heating events can release Fe and Ni into the gas phase and subsequently regenerate nanometre-scale kamacite through rapid recondensation. Mild shocks observed in returned Ryugu samples (∼2 GPa, <500 °C) further demonstrate that shock processing can mobilise metals without causing extensive dehydration or melting, enabling shock-formed nanophase kamacite to persist in hydrated asteroidal and cometary materials.
3. In broader terms, the results depict a scenario where low-velocity shocks (1–2 km/s) in the ISM, protoplanetary disks, and cometary environments serve as efficient factories for metal nanoparticles. These particles are later oxidised, embedded in amorphous silicates, or transported by radiation pressure into the diffuse ISM. Shock-induced formation of nanophase kamacite thus becomes a crucial process connecting metal vapor chemistry, dust evolution, and the cycling of refractory elements from stellar ejecta through interstellar space into the earliest solids of planetary systems.

## Data Availability

The original contributions presented in the study are included in the article/Supplementary Material, further inquiries can be directed to the corresponding author.
