# Supplementary material for: Shock-induced nucleation of nanophase Fe-Ni alloy and its implications for interstellar iron reservoirs
Source: Front Chem. 2026 Feb 20;14:1774797. doi: 10.3389/fchem.2026.1774797 (PMC12962654; doi:10.3389/fchem.2026.1774797)
Supplement: Supplementary file 1 [file DataSheet1.pdf]

Shock-Induced Nucleation of Nanophase Fe-Ni Alloy and Its Implications for Interstellar Iron Reservoirs

Prakash Velampatti Selvaraj[1], Vijayanand Chandrasekaran[1,2*]

[1]Department of Chemistry, School of Advanced Sciences, Vellore Institute of Technology, Vellore, Tamil Nadu-632 014, India

[2]Department of Analytics, School of Computer Science and Engineering, Vellore Institute of Technology, Vellore, Tamil Nadu-632 014, India

***Correspondence:** Vijayanand Chandrasekaran
vijayanand.c@vit.ac.in

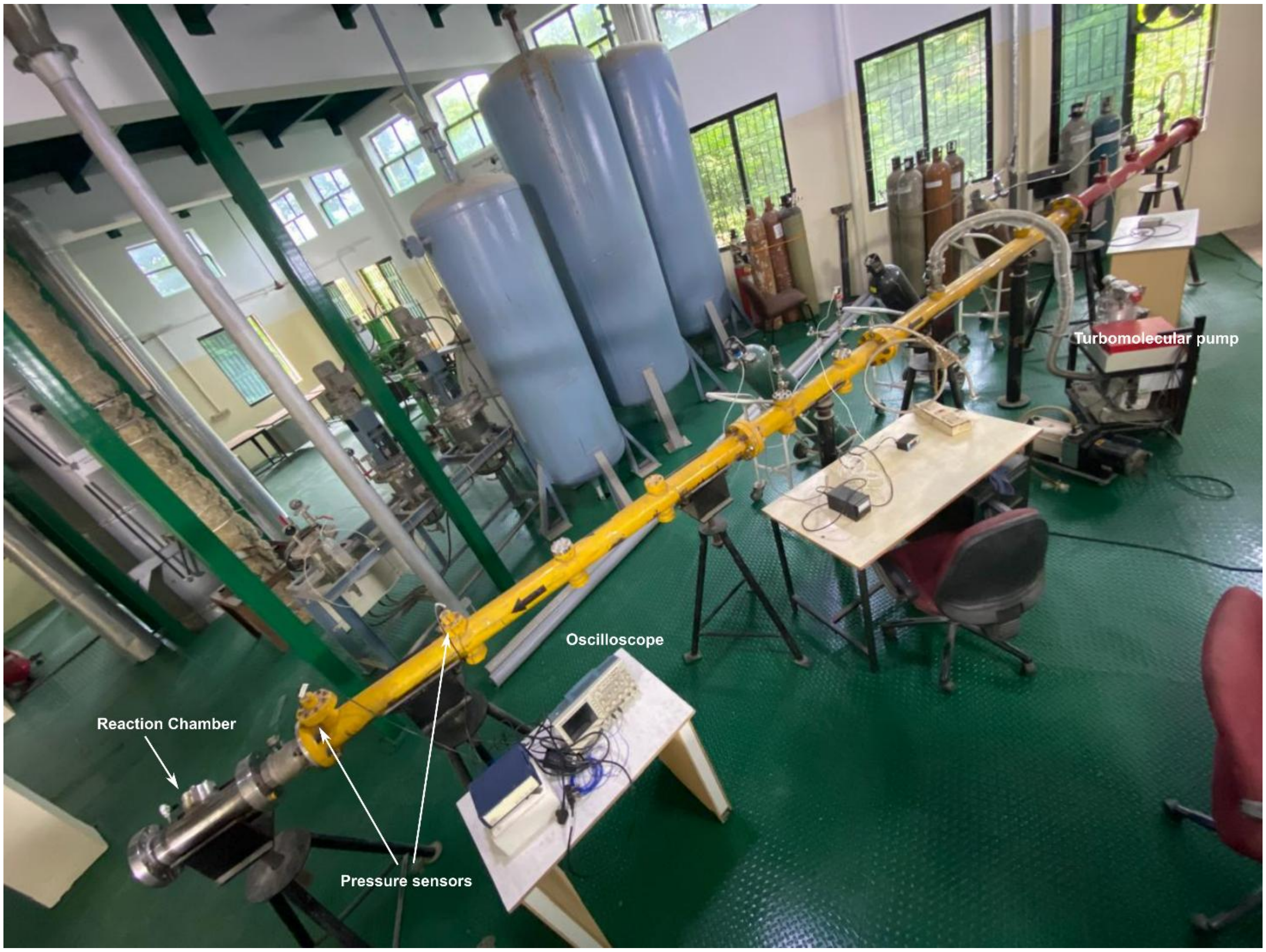

Supplementary Figure S1. Photographic image of laboratory shock tube setup

2. Powder X-Ray Diffraction Analysis

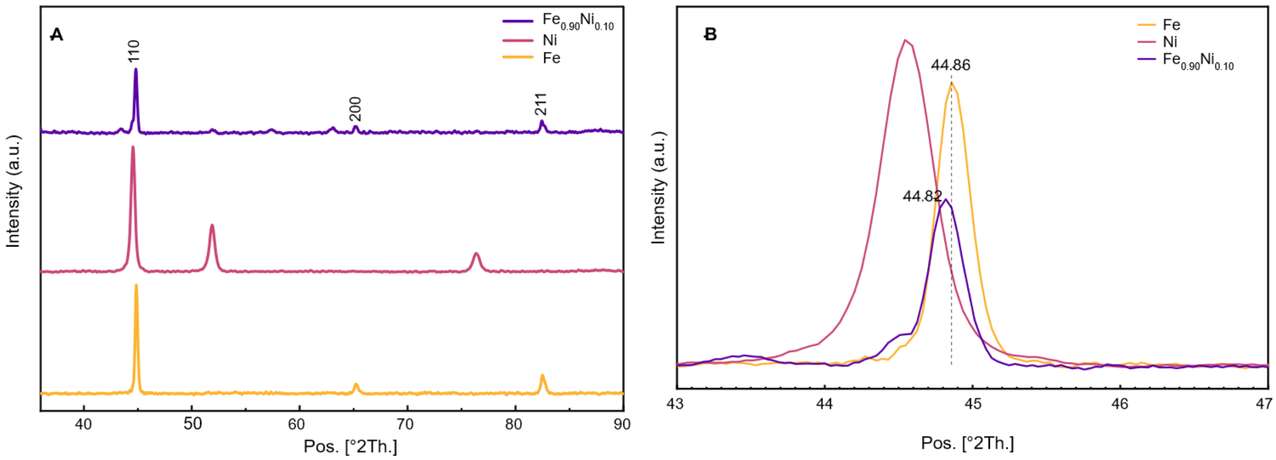

Supplementary Figure S2. (**A, B**) X-ray diffraction (XRD) patterns of synthesized kamacite (purple), pure iron (yellow), and pure nickel (pink), illustrating peak shifts and phase identification.

3. FESEM with EDS Analyses

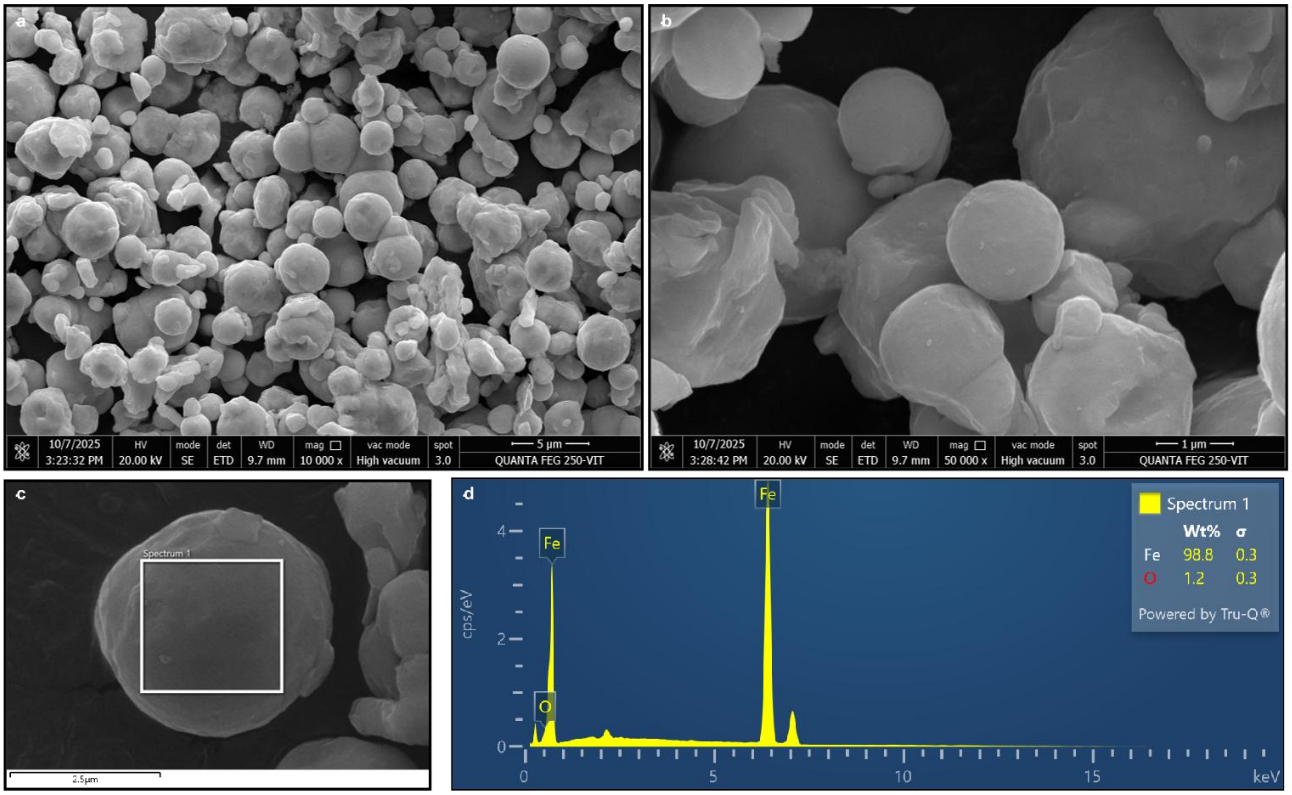

Supplementary Figure S3. Secondary electron images and EDS of pure iron. (A, B) Secondary electron (SE) images of pure Fe. (**C, D**) SE image of a representative Fe (spherical) particle and corresponding EDS spectrum. The minor oxygen signal in the EDS spectrum is attributed to residual vacuum or chamber contamination rather than intrinsic oxidation of the Fe standard.

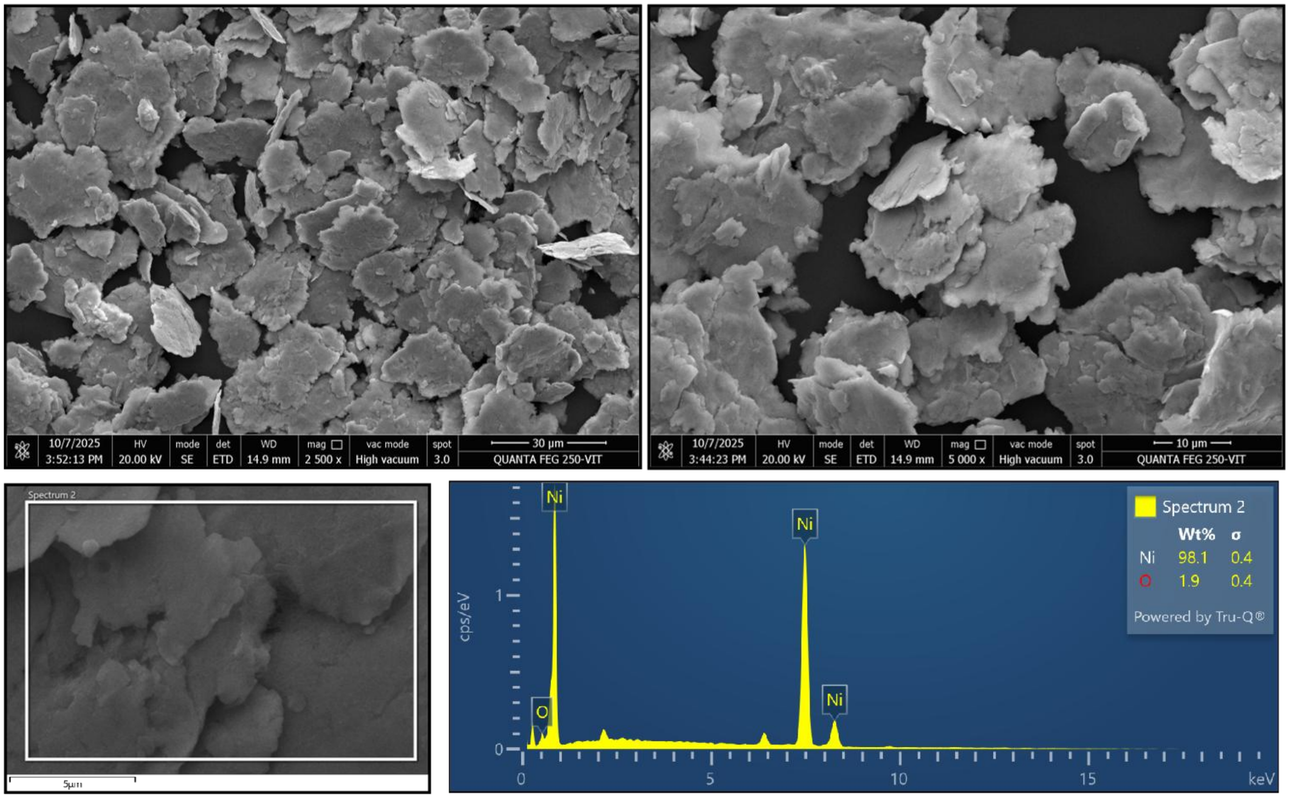

Supplementary Figure S4. Secondary electron images and EDS of pure nickel. (**A, B**) Secondary electron (SE) images of pure Ni. (**C, D**) SE image of a representative Ni flake and corresponding EDS spectrum. The minor oxygen signal in the EDS spectrum is attributed to residual vacuum or chamber contamination rather than intrinsic oxidation of the Ni standard.

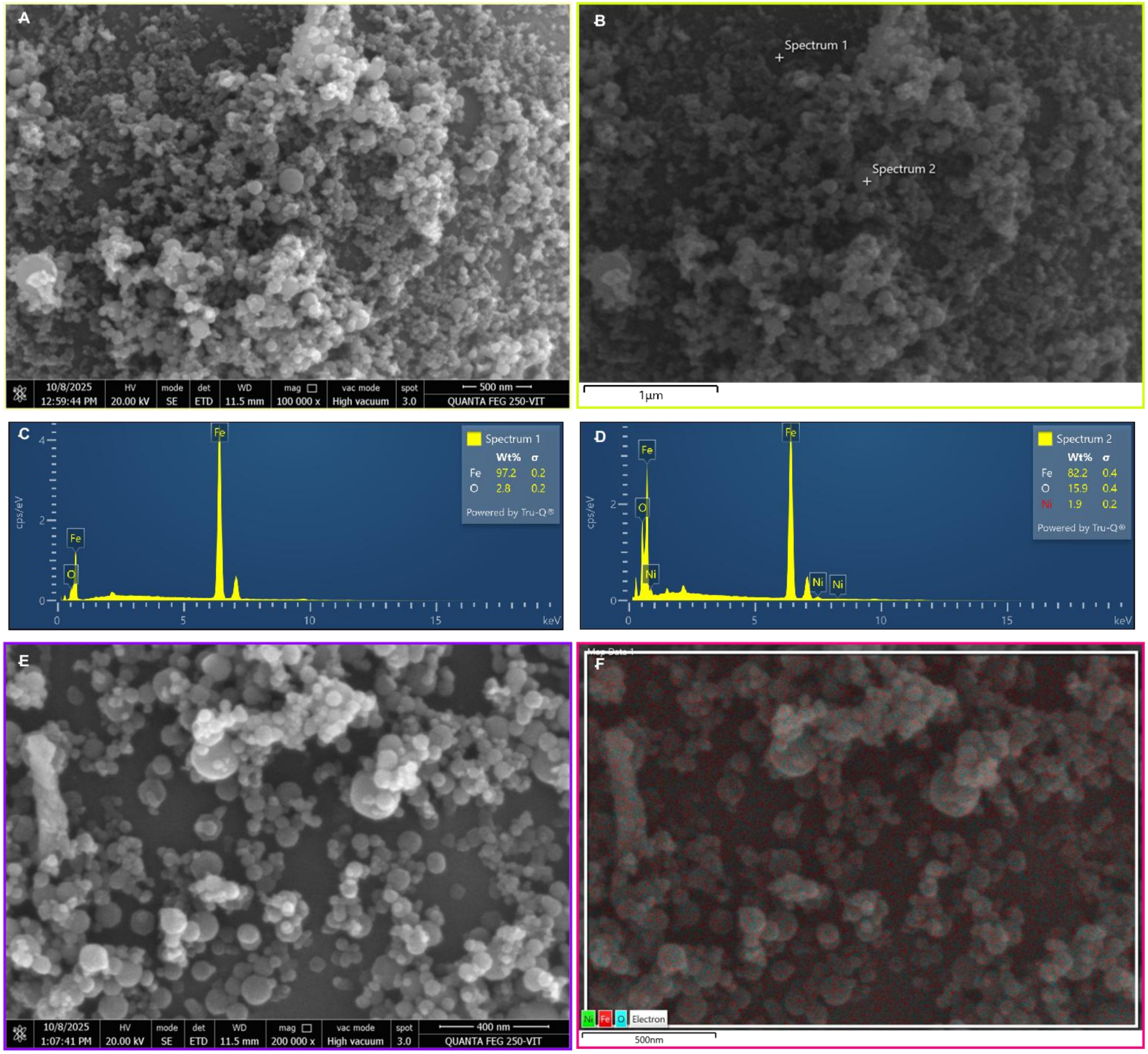

Supplementary Figure S5. FESEM, point EDS and elemental mapping of shock-processed Sample-1 ($Fe_{0.90}Ni_{0.10}$). (**A and E**) Secondary electron (SE) images showing kamacite grains with characteristic octagonal to rounded morphologies and sharp boundaries relative to surrounding phases. (**B–D**) Point energy-dispersive X-ray spectroscopy (EDS) spectra: spectrum 1 identifies an iron oxide, whereas analysis of an octagonal grain confirms kamacite, with strong Fe and Ni peaks and only minor oxygen is attributed to residual vacuum. (**F**) EDS elemental maps corresponding to E, showing the spatial distributions of Fe and Ni within the kamacite domains and confirming Fe–Ni alloy enrichment and compositional homogeneity.

3 Transmission Electron Microscopy Analyses

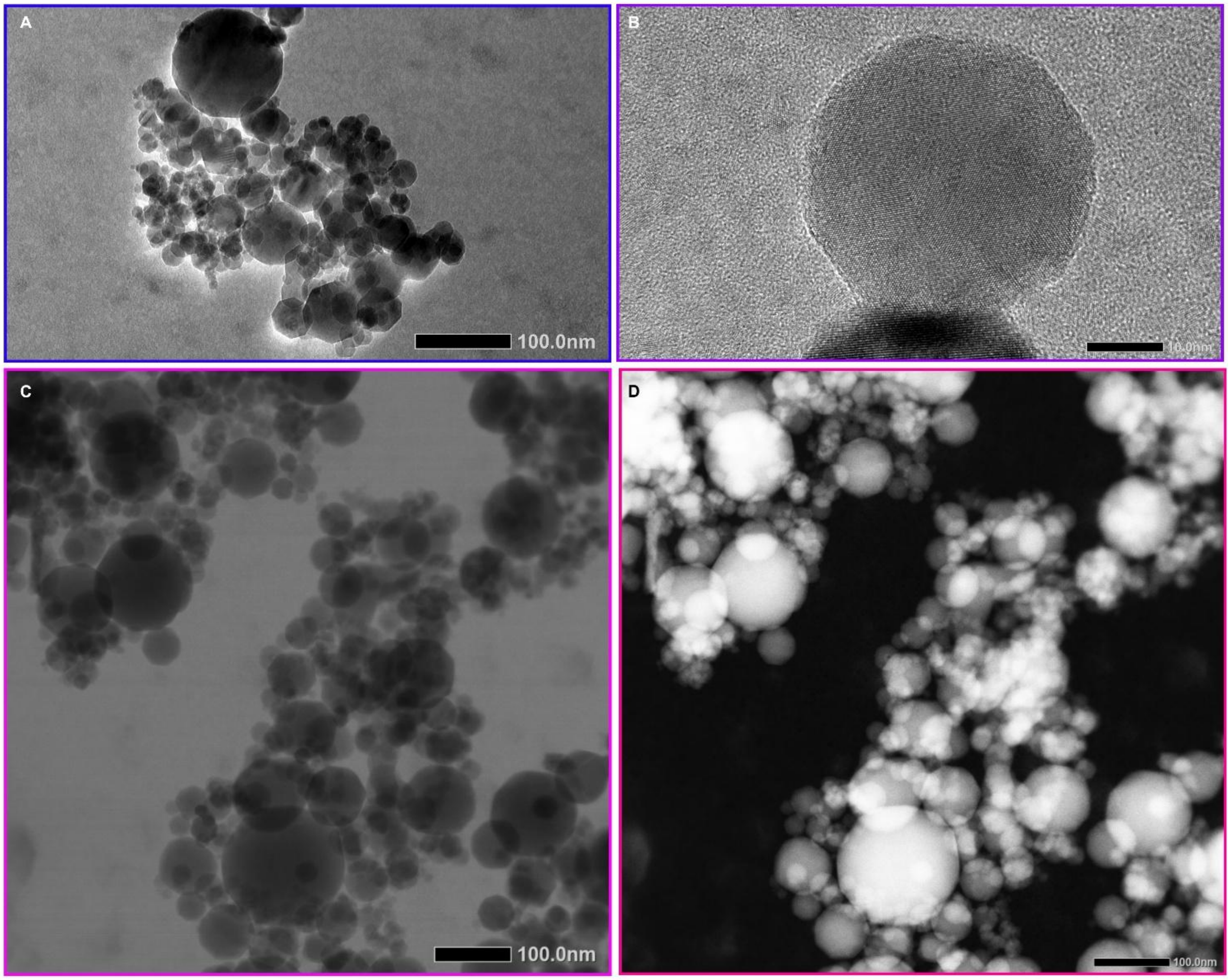

Supplementary Figure S6. TEM and STEM morphology of shock-synthesized kamacite nanoparticles. (A, B) TEM and HRTEM images showing the morphology of nanophase kamacite. **(C, D)** STEM–BF and HAADF images of shock-synthesized sample-1 (Fe–Ni) illustrating kamacite nanoparticles with octagonal to spherical shapes.

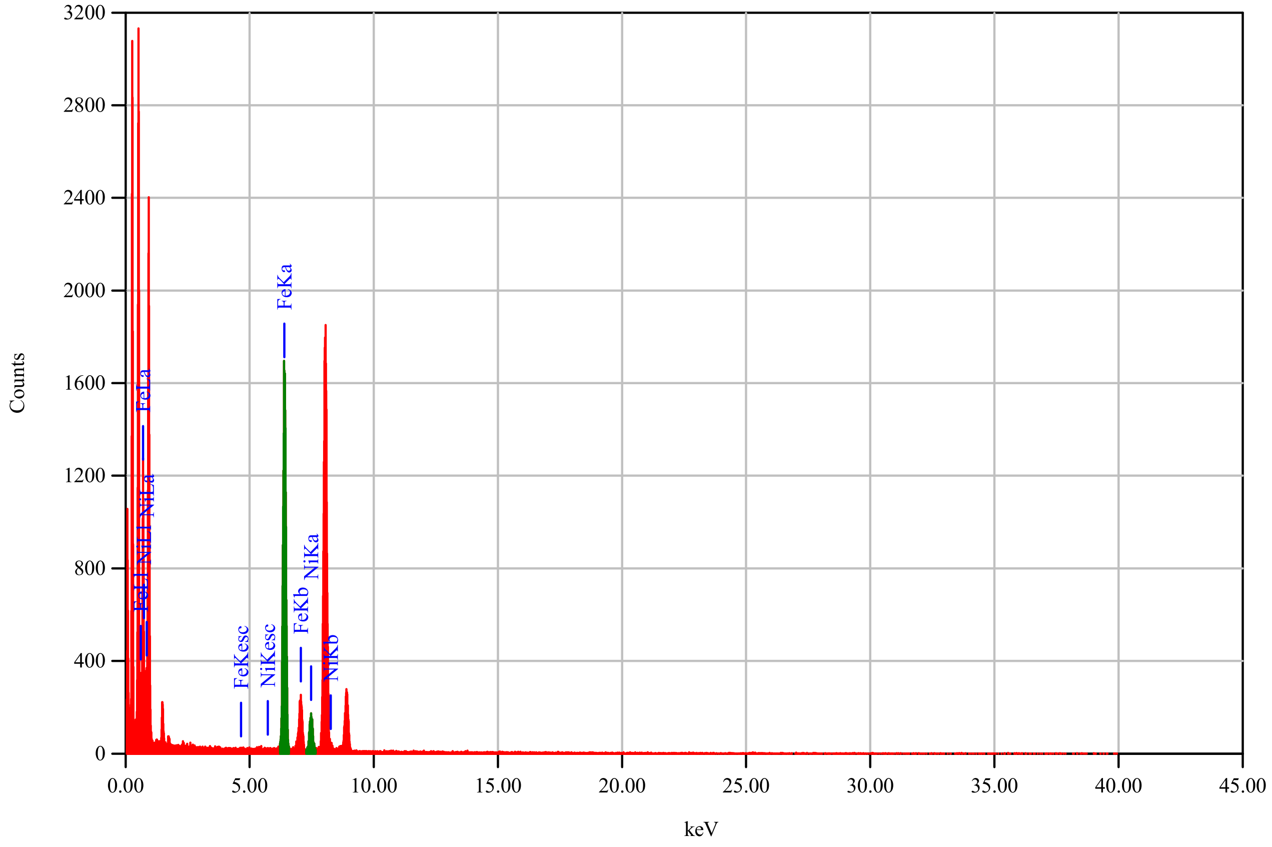

Element	(keV)	Mass%	Counts	Sigma	Atom%	Compound	Mass%	Cation	K
Fe K (Ref.)	6.398	90.54	22841.08	1.28	90.96				1.0000
Ni K	7.471	9.46	2109.12	0.45	9.04				1.1316
Total		100.00			100.00				

Supplementary Figure S7. Quantitative EDS analysis of kamacite grains. Quantitative EDS analysis of the mapped grains in **Fig. 6G** yields average Fe and Ni contents of 90.96 and 9.04 atomic percent (at.%), respectively.

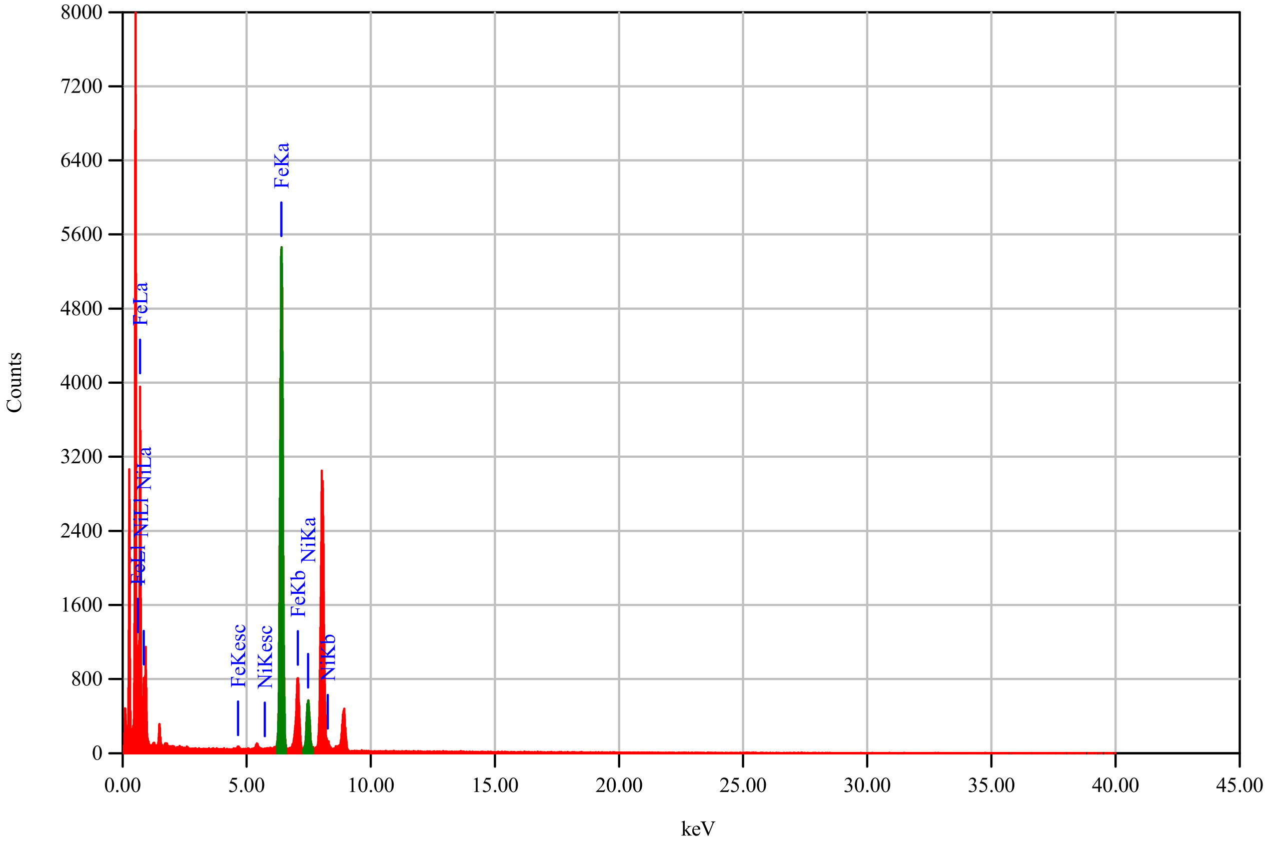

Element	(keV)	Mass%	Counts	Sigma	Atom%	Compound	Mass%	Cation	K
Fe K (Ref.)	6.398	89.62	74645.71	0.70	90.08				1.0000
Ni K	7.471	10.38	7636.47	0.26	9.92				1.1316
Total		100.00			100.00				

Supplementary Figure S8. Quantitative EDS analysis of kamacite grains. Quantitative EDS analysis of the mapped grains in **Fig. 7A** yields average Fe and Ni contents of 90.08 and 9.92 atomic percent (at.%), respectively.
